# BioGraphBench-PPI enables audit-first evaluation of negative-sampling contracts and structural baselines in protein interaction networks

**DOI:** 10.3389/fbinf.2026.1966469

**Published:** 2026-10-01

**Authors:** Alejandro Cabrera-Andrade, Jorge Robalino-Díaz, Iván Dueñas-Espín, Ana Karina Zambrano, William Villegas-Ch

**Affiliations:** 1 Grupo de Bio-Quimioinformática, Universidad de Las Américas, Quito, Ecuador; 2 Escuela de Enfermería, Facultad de Ciencias de La Salud, Universidad de Las Américas, Quito, Ecuador; 3 Escuela de Ingeniería en Ciberseguridad, Facultad de Ingeniería y Ciencias Aplicadas, Universidad de Las Américas, Quito, Ecuador; 4 Grupo de Investigación en Inequidades y Determinantes Ecológicos y Ambientales en Salud, Instituto de Salud Pública, Facultad de Salud y Bienestar, Pontificia Universidad Católica del Ecuador, Quito, Ecuador; 5 Facultad de Ciencias de La Salud Eugenio Espejo, Centro de Investigación Genética y Genómica, Universidad UTE, Quito, Ecuador

**Keywords:** benchmarking, biomedical networks, calibration, link prediction, negative sampling, node2vec, protein-protein interactions, reproducibility

## Abstract

**Introduction:**

Biomedical graph-learning results are difficult to compare when source versions, identifier rules, data splits, structural features, and sampled-nonedge contracts are incompletely documented.

**Methods:**

We developed BioGraphBench-PPI as an audit-first benchmark for protein-protein interaction (PPI) link prediction using STRING v12.0, BioGRID 5.0.260, and two cross-resource no-overlap ablations. For each PPI task, we generated 10 predefined seeds and three balanced sampled-nonedge contracts: random, degree-matched, and two-hop. We compared local heuristics, logistic regression, random forest, histogram gradient boosting (HGB), an auditable node2vec-compatible random-walk baseline, and a two-layer GCN link-prediction baseline, reporting ranking, calibration, runtime, and confidence intervals.

**Results:**

Random sampled nonedges yielded high HGB AUPRC values (0.942–0.964), but degree-matched sampled nonedges reduced AUPRC by 0.049–0.259. The GCN baseline showed signal under random negatives in selected graphs (AUPRC 0.526–0.717) but approached chance under degree-matched and two-hop contracts (AUPRC 0.500–0.515).

**Discussion:**

BioGraphBench-PPI shows that PPI benchmark conclusions depend strongly on sampled-nonedge design and graph source. The benchmark therefore contributes an auditable evaluation contract rather than a claim that PPI prediction is solved.

## 1 Introduction

Biomedical knowledge is naturally relational: proteins interact, genes participate in pathways, compounds bind targets, and phenotypes emerge from connected biological processes. Graph neural networks (GNNs) offer a flexible way to combine this relational structure with molecular or functional attributes, and they now support applications ranging from protein–protein interaction (PPI) prediction to therapeutic discovery (Sun et al., 2020; Yue et al., 2020; Huang et al., 2022; Jha et al., 2022). At the same time, broad benchmarking initiatives have shown that progress in graph learning depends on more than architectural novelty. Open Graph Benchmark, MoleculeNet, and systematic GNN benchmarking have emphasized shared datasets, explicit splits, and comparable evaluation (Wu et al., 2018; Hu et al., 2020; Dwivedi et al., 2023). Biomedical initiatives such as OpenBioLink and the Open Biomedical Network Benchmark (OBNB) extend this logic to heterogeneous biomedical graphs and biological network tasks (Breit et al., 2020; Liu and Krishnan, 2024).

A persistent gap remains between the availability of biomedical networks and their use as controlled machine-learning benchmarks. STRING and BioGRID are high-value, openly accessible resources for protein associations and curated interactions (Oughtred et al., 2021; Szklarczyk et al., 2023), but they were not designed as closed experimental datasets. Their records may differ in evidence, coverage, identifiers, redundancy, and release cadence, and the same protein pair may occur in both resources. A benchmark built without an explicit audit can therefore reward accidental data availability, cross-resource reuse, or an overly permissive split. Evaluation design is especially important for GNNs because message passing can propagate information that should be unavailable at test time, and apparently minor differences in splitting or feature construction can materially alter conclusions (Shchur et al., 2018; Dwivedi et al., 2023).

Recent Frontiers contributions in network bioinformatics illustrate the editorial emphasis on usable tools, transparent algorithms, and reproducible validation. These include an interactive protein-contact network explorer (Ganapathy et al., 2026), a nonlinear gene co-expression method (Bacong et al., 2026), a community-based drug-repositioning pipeline (Colibasanu et al., 2025), and a notebook-centered reproducible systems-biology workflow (Liebal et al., 2021). Closely related PPI prediction studies also use stratified repeated evaluation and controlled comparisons to assess whether graph architectures add value (Ebeid et al., 2025). BioGraphBench addresses the same methodological space from a benchmark-design perspective: its primary object is the auditable evidence chain that makes model comparisons credible across biological network resources.

Biomedical graph applications span drug repurposing, molecular interaction prediction, combination therapy, and knowledge mapping (Himmelstein et al., 2017; Sun et al., 2020; Besharatifard and Vafaee, 2024; Yao et al., 2024). Foundational embedding methods such as node2vec remain relevant controls when assessing gains from graph neural networks (Grover and Leskovec, 2016). This breadth increases the need for benchmark contracts that separate gains from data reuse, feature leakage, and favorable negative sampling.

PPI link prediction illustrates this problem. Numerous graph-based models report high predictive performance (Huang et al., 2020; Jha et al., 2022; Zhou et al., 2022; Zeng et al., 2024), but topology alone can be a strong discriminator: common-neighbor scores, degree products, and their supervised combinations may already separate observed interactions from randomly sampled non-edges. Without these controls, improvement over a weak baseline is difficult to interpret as additional biological learning. A complementary challenge appears in protein function annotation, where labels are sparse and highly imbalanced. In that setting, area under the receiver operating characteristic curve (AUROC) can appear acceptable while precision–recall performance and thresholded predictions remain poor. Ranking, calibration, and decision metrics must therefore be reported together.

We present BioGraphBench-PPI, an audit-first experimental benchmark and methodological workflow for reproducible learning on protein interaction networks. The central research question is whether open PPI resources can be transformed into link-prediction tasks whose evidence chain is reconstructible from source download through canonicalization, split generation, feature construction, model fitting, and statistical reporting.

The contribution is both methodological and empirical. Methodologically, the benchmark makes data provenance, leakage control, negative sampling, and split contracts first-class experimental objects rather than *post hoc* documentation. Empirically, it establishes a demanding multi-seed non-GNN floor for PPI link prediction and shows that model ranking depends on the negative-sampling contract.

## 2 Materials and methods

### 2.1 Audit-first benchmark design

BioGraphBench treats each dataset–task combination as a versioned experimental object

$$B_{k}=\left(G_{k},T_{k},S_{k},\Phi_{k},M_{k}\right),G_{k}=\left(V_{k},E_{k},X_{k},Y_{k}\right)$$

Here, k indexes a dataset-task instance. G_k_ denotes the canonical graph for task k; T_k_ is the prediction task; S_k_ contains the predefined partitions and sampled-nonedge contracts; Φ_k_ specifies the feature-construction policy; and M_k_ defines the metric set. Within G_k_, V_k_ is the node set, E_k_ the edge set, X_k_ the input representation, and Y_k_ the labels or prediction targets. An experiment is accepted only when its source, transformation rules, splits, sampled-nonedge contract, features, model configuration, seed, and outputs have machine-readable artifacts. Figure 1 summarizes the BioGraphBench-PPI evidence chain.

**FIGURE 1:**
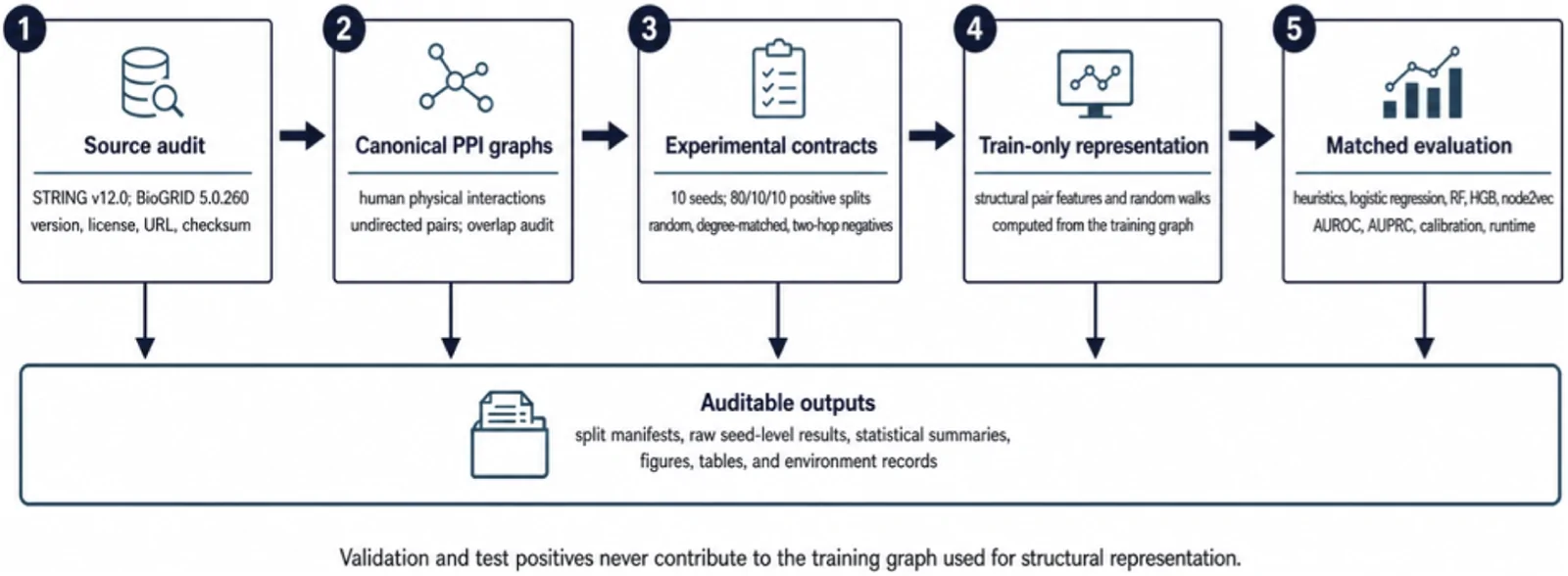
Audit-first BioGraphBench workflow. Open biological resources are audited and canonicalized before positive partitions and sampled-nonedge contracts are defined. Structural features and random-walk representations are derived exclusively from the training-positive graph, followed by matched evaluation of discrimination, calibration, and runtime. The lower band summarizes auditable outputs. AUPRC, area under the precision-recall curve; AUROC, area under the receiver operating characteristic curve; HGB, histogram gradient boosting; PPI, protein-protein interaction; RF, random forest.

Candidate datasets were screened for reproducible bulk access, licensing or usage terms, schema clarity, identifier interpretability, and suitability for a graph-learning task. Every acquired file has a manifest containing the source URL, observed version, acquisition date, byte size, and SHA-256 checksum. Raw data are immutable inputs; derived data and reports are stored separately. The snapshot used for this manuscript also contains the task contracts, result tables, analysis scripts, and tests required to trace each reported quantity.

### 2.2 Data sources and canonicalization

The first PPI source was the human physical-interaction network from STRING version 12.0, acquired on 4 August 2026 (Szklarczyk et al., 2023). All physical-interaction records were retained without a confidence-score threshold, and combined_score was preserved as edge metadata. The second source was the human subset of BioGRID release 5.0.260, observed on the same date (Oughtred et al., 2021). BioGRID records were restricted to physical interactions for which both endpoints corresponded to *Homo sapiens* (taxonomy identifier 9,606) and had valid Entrez Gene identifiers. STRING protein identifiers were mapped to Entrez Gene identifiers for the cross-resource comparison described below. For both sources, an interaction (u,v) was converted to the canonical undirected pair

$$\mathrm{canon}\left(u,v\right)=\left(\min\left(u,v\right),\max\left(u,v\right)\right)$$

after excluding self-interactions and records without valid endpoint identifiers; duplicate canonical pairs were collapsed.

The secondary task used OBNB data package version 0.1.0 (Liu and Krishnan, 2024). Its BioGRID network contains 19,765 nodes and 1,554,790 undirected edges. The resulting multilabel task comprised 114 GOBP labels. The partially observed label matrix contained 10,199 positive labels among 692,550 observed node–label entries. OBNB’s publication-count-based ratio partition assigned 3,645 labeled nodes to training, 1,215 to validation, and 1,215 to testing.

### 2.3 Task definitions and fixed partitions

Five tasks were retained in the benchmark, with four PPI link-prediction tasks forming the central contribution of this manuscript (Table 1). Their graph sizes and positive-partition sizes are summarized in Table 2. For each PPI graph, positive edges were shuffled with 10 predefined seeds (42–51) and partitioned into 80% training, 10% validation, and 10% testing subsets while preserving a spanning forest in the training graph. The OBNB task is retained as a secondary stress-test artifact, while the quantitative claims of the present study are centered on PPI link prediction.

$$E^{+}=E^{+}\mathrm{train}⊎E^{+}\mathrm{val}⊎E^{+}\mathrm{test}$$

**TABLE 1:** Accepted tasks in the BioGraphBench benchmark framework. The present study centers on the four PPI link-prediction tasks; OBNB is retained as a secondary stress-test artifact.

Task	Source	Prediction unit	Primary metrics	Role
STRING physical	STRING v12.0	Undirected protein pair	AUROC, AUPRC	Main PPI
BioGRID physical	BioGRID 5.0.260	Undirected protein pair	AUROC, AUPRC	Main PPI
BioGRID without STRING	BioGRID after pair removal	Undirected protein pair	AUROC, AUPRC	Overlap ablation
STRING without BioGRID	STRING after pair removal	Undirected protein pair	AUROC, AUPRC	Overlap ablation
OBNB BioGRID + GOBP	OBNB 0.1.0	Protein with 114-label vector	Macro-AUROC, macro-AUPRC	Secondary challenge

**TABLE 2:** Scale and fixed positive partitions of the accepted tasks. PPI counts are positive edges per seed; each partition is paired with an equal number of negatives under each PPI negative-sampling contract.

Task	Nodes	Graph edges	Train	Validation	Test
STRING physical	18,767	738,805	591,045	73,880	73,880
BioGRID physical	20,376	961,531	769,225	96,153	96,153
BioGRID without STRING	19,591	538,003	430,403	53,800	53,800
STRING without BioGRID	16,781	314,539	251,633	31,453	31,453
OBNB BioGRID + GOBP	19,765	1,554,790	3,645	1,215	1,215

All structural features were computed from Gtrain = (V, E + train) only. For each PPI partition and seed, three balanced sampled-nonedge contracts were produced. The random contract samples uniformly from unordered node pairs absent from the complete observed positive-edge set. The degree-matched contract approximately preserves the endpoint log-degree-bin distribution of the corresponding positive partition. The two-hop contract samples unobserved pairs whose endpoints share at least one neighbor in the training-positive graph, creating a local hard-negative condition without using validation or test positives to define hardness. Across all three contracts, sampled nonedges were required to be absent from the complete observed positive-edge set, and the training, validation, and test sampled-nonedge sets were mutually exclusive.

To quantify direct reuse between sources, interaction pairs were aligned in Entrez Gene space for cross-resource comparison. Overlap statistics were calculated on aligned Entrez pair sets, whereas STRING ablation was counted in the original STRING protein-pair edge space. The audit produced the following counts: 738,805 original STRING edges; 7,623 STRING edges without complete Entrez mapping; 16,659 STRING edges with ambiguous Entrez mappings; 766,284 Entrez pairs after explicit STRING-to-Entrez expansion; 423,528 unique Entrez pairs overlapping BioGRID; and 424,266 original STRING protein-pair edges removed because at least one expanded Entrez pair overlapped BioGRID. Thus, the 738-edge gap is not a numerical inconsistency: it reflects the difference between unique Entrez-space overlap counting and source-edge-space removal after explicit identifier expansion. For sources a and b,

$$E\left(a\setminus b\right)=\left\{e\in E\left(a\right):\;e\notin E\left(b\right)\right\}$$

This procedure controls direct pair overlap but does not establish complete biological independence, because the resources can still share nodes, experimental literature, and higher-order topology.

The fifth task is multilabel node classification. Each evaluation unit is a protein and its 114-dimensional binary GOBP label vector. Labels were never included in input features. Model selection and decision-threshold selection used validation data only; the test partition was reserved for final evaluation.

### 2.4 Structural features and link-prediction baselines

For a candidate pair (u,v), the structural feature vector contained common neighbors (CN), Jaccard coefficient, Adamic–Adar (AA), preferential attachment (PA), and the two endpoint degrees:

$$\varphi \left(u,v\right)=\left[\text{CN},\text{Jaccard},\text{AA},\text{PA},\deg\left(u\right),\deg\left(v\right)\right]$$

Each individual heuristic served as an unsupervised score. The complete feature vector was supplied to logistic regression, random forest, and histogram gradient boosting (HGB) as supervised structural baselines. We additionally implemented an auditable node2vec-compatible random-walk baseline using unbiased walks (p = q = 1) and a logistic edge decoder trained on dot product, cosine similarity, L1 distance, and L2 distance features. Finally, we evaluated a two-layer GCN link-prediction baseline. The GCN propagation graph used only training-positive edges; node inputs were constant, degree, log-degree, and one-hot log-degree-bin features derived from that training graph; and candidate pairs were scored with a dot-product decoder. The GCN was trained for 10 epochs with up to 100,000 positive and 100,000 sampled-negative training pairs per seed, while evaluation used the predefined validation and test splits. Models were implemented with scikit-learn and PyTorch, and all model configurations and seeds are recorded in output manifests.

### 2.5 Multilabel node-classification baselines

The node-classification input was a one-hot encoding of logarithmic degree bins, compared with a constant-feature logistic regression control. We evaluated one-vs-rest logistic regression, a multilayer perceptron (MLP), and a two-layer graph convolutional network (GCN). The neural pilots were implemented with PyTorch 2.4.1 on CPU. Binary cross-entropy was applied across observed node–label entries. Because a single global 0.5 threshold yielded nearly no positive decisions, per-label thresholds for the one-hot degree logistic regression, MLP, and GCN models were selected using validation predictions and then applied unchanged to the test set. These neural results assess pipeline functionality and task difficulty; they are not a comprehensive GNN architecture search.

### 2.6 Evaluation, calibration, and reproducibility

For link prediction, test AUROC and AUPRC measured ranking discrimination. Probability quality was assessed with Brier score and expected calibration error using 10 equally spaced confidence bins (ECE-10):

$$\text{ECE}=\sum_{b=1}^{B}\frac{\left|I_{b}\right|}{n}\left|\text{acc}\left(I_{b}\right)-\text{conf}\left(I_{b}\right)\right|,B=10$$

Training wall-clock time was recorded as a descriptive computational measure on the local CPU execution environment used for the benchmark: Intel Core i9-13900HX, 24 physical cores, 32 logical processors, PyTorch CPU execution for neural pilots, and the pinned Python package versions reported above. For multilabel node classification, we report macro-AUROC and macro-AUPRC together with threshold-dependent micro-F1, micro-precision, and micro-recall where available; decision thresholds were selected using validation data only.

For each PPI dataset-contract-model combination, performance metrics were summarized as the mean and sample standard deviation across 10 seeds. Ninety-five percent confidence intervals for the corresponding means were calculated using the Student t distribution with 9 degrees of freedom. Effects of the sampled-nonedge contract were evaluated as seed-paired differences between random and degree-matched conditions and between random and two-hop conditions, using the same seed in each comparison. Paired Wilcoxon signed-rank tests and paired standardized effect sizes were computed to support contract-level comparisons.

The software environment was pinned to NumPy 1.24.3, pandas 2.0.3, SciPy 1.10.1, scikit-learn 1.3.2, PyTorch 2.4.1+CPU, and OBNB 0.1.0. Data checks, split validation, feature-policy tests, artifact-presence tests, raw per-seed outputs, statistical summaries, and regenerated figure/table scripts were retained with the benchmark. The central PPI results in the present benchmark release come from the completed 10-seed protocol; the OBNB node-classification experiment is retained as a secondary challenge analysis.

## 3 Results

### 3.1 Accepted tasks and benchmark scale

The accepted task set should be read as a quality-control result, not only as a list of available datasets. BioGraphBench retained five auditable tasks because their source files, transformations, split definitions, feature policies, and outputs could be reconstructed from local manifests. Table 1 separates the central PPI contribution from the retained OBNB stress test. Four tasks evaluate undirected PPI link prediction, whereas OBNB represents a distinct secondary node-labeling task with a different prediction target and performance regime.

This distinction is important for defining the scope of the study. The present manuscript centers its statistical evidence on PPI link prediction, while OBNB is retained as a secondary stress-test task that demonstrates the applicability of the audit framework to a distinct biological graph-learning problem. Table 1 therefore serves two purposes. It documents the broader benchmark scaffold and clearly delineates the empirical scope of the analyses reported here.

The scale summary in Table 2 shows why the PPI study is a meaningful stress test even before model comparison. The complete STRING and BioGRID graphs are not small illustrative examples; they contain hundreds of thousands of canonical interactions. The no-overlap ablations are also large enough to support evaluation, but they remove shared cross-resource pairs that could otherwise make performance appear more general than it is. This makes the ablations essential rather than decorative: they test whether a model is learning robust structural regularities or benefiting from database overlap.

Across four PPI datasets, three sampled-nonedge contracts, and 10 seeds, the protocol generated 120 split manifests. The multi-seed design enabled assessment of sampling stability in addition to deterministic reproducibility. These runs produced 1,680 classical-baseline result rows, 240 node2vec-compatible result rows, and 240 GCN link-prediction result rows, providing the empirical basis for confidence intervals, paired seed-wise comparisons, calibration summaries, runtime summaries, and model-ranking analyses.

### 3.2 Strong structural baselines for PPI link prediction

Table 3 summarizes contract-aware performance across the four PPI datasets and five model families. Under random negatives, the supervised structural models perform strongly, especially HGB and random forest. HGB reaches mean test AUPRC values of 0.950 for STRING, 0.943 for BioGRID, 0.964 for STRING without BioGRID, and 0.942 for BioGRID without STRING. If this were the only result, the benchmark would appear nearly solved by local topology.

**TABLE 3:** Mean test AUPRC across 10 seeds by PPI dataset, negative-sampling contract, and model family, including the GCN link-prediction baseline.

Task	Negatives	LogReg	RF	HGB	node2vec	GCN
STRING	Random	0.946	0.950	0.950	0.877	0.535
STRING	Degree-matched	0.864	0.881	0.881	0.804	0.500
STRING	Two-hop	0.753	0.857	0.856	0.731	0.501
BioGRID	Random	0.935	0.942	0.943	0.873	0.526
BioGRID	Degree-matched	0.716	0.771	0.770	0.698	0.500
BioGRID	Two-hop	0.654	0.773	0.774	0.639	0.500
STRING without BioGRID	Random	0.960	0.964	0.964	0.941	0.717
STRING without BioGRID	Degree-matched	0.889	0.915	0.916	0.904	0.500
STRING without BioGRID	Two-hop	0.682	0.851	0.847	0.736	0.515
BioGRID without STRING	Random	0.933	0.941	0.942	0.898	0.591
BioGRID without STRING	Degree-matched	0.594	0.687	0.684	0.725	0.500
BioGRID without STRING	Two-hop	0.722	0.830	0.849	0.605	0.501

The same table shows why that interpretation would be misleading. Degree-matched negatives reduce the shortcut provided by endpoint degree, while two-hop negatives force discrimination among locally plausible pairs. The model rankings are no longer invariant: HGB and random forest remain strong in most contexts, node2vec becomes competitive in the BioGRID-without-STRING degree-matched condition, and random forest slightly exceeds HGB for STRING-without-BioGRID under two-hop negatives. The GCN baseline adds a neural control to this pattern. It shows non-trivial signal under random negatives in the two no-overlap ablations, especially STRING without BioGRID (AUPRC 0.717), but collapses toward chance under degree-matched and two-hop negatives (AUPRC approximately 0.500–0.515). This is a methodological result, not just a model result, because benchmark conclusions change when the negative contract changes.

Table 4 quantifies the cost of replacing random negatives with harder contracts. Degree-matched negatives reduce HGB AUPRC by 0.069 in STRING, 0.172 in BioGRID, 0.049 in STRING without BioGRID, and 0.259 in BioGRID without STRING. The largest drop occurs in BioGRID without STRING, which is precisely the setting where overlap has been removed and degree matching most strongly challenges the structural baseline.

**TABLE 4:** HGB sensitivity to negative-sampling contracts. Drops are seed-averaged AUPRC differences relative to random negatives.

Task	Random HGB	Degree HGB	Two-hop HGB	Drop random-degree	Drop random-two-hop
STRING	0.950	0.881	0.856	0.069	0.094
BioGRID	0.943	0.770	0.774	0.172	0.169
STRING without BioGRID	0.964	0.916	0.847	0.049	0.117
BioGRID without STRING	0.942	0.684	0.849	0.259	0.093

The two-hop contract tells a complementary story. Its performance loss relative to random negatives is substantial in STRING and STRING without BioGRID, but it does not behave identically to degree matching. In BioGRID without STRING, two-hop HGB AUPRC rises to 0.849, higher than the degree-matched value of 0.684. This reversal is important: hard negatives are not interchangeable. Degree matching controls endpoint popularity, whereas two-hop sampling emphasizes local closure. A defensible benchmark should report both because they test different failure modes.

Figure 2 shows that the PPI results are not driven by unstable seed behavior. In Figure 2A, the complete STRING and BioGRID graphs show extremely narrow HGB distributions across 10 seeds. STRING remains highest under random negatives (mean AUPRC 0.950), decreases under degree-matched negatives (0.881), and decreases further under two-hop negatives (0.856). BioGRID shows a sharper random-to-degree transition, falling from 0.943 to 0.770, while the two-hop condition remains similar to degree matching at 0.774. This panel therefore separates two issues: STRING is consistently easier than BioGRID under the harder contracts, and the seed variance is small relative to the protocol-induced shift.

**FIGURE 2:**
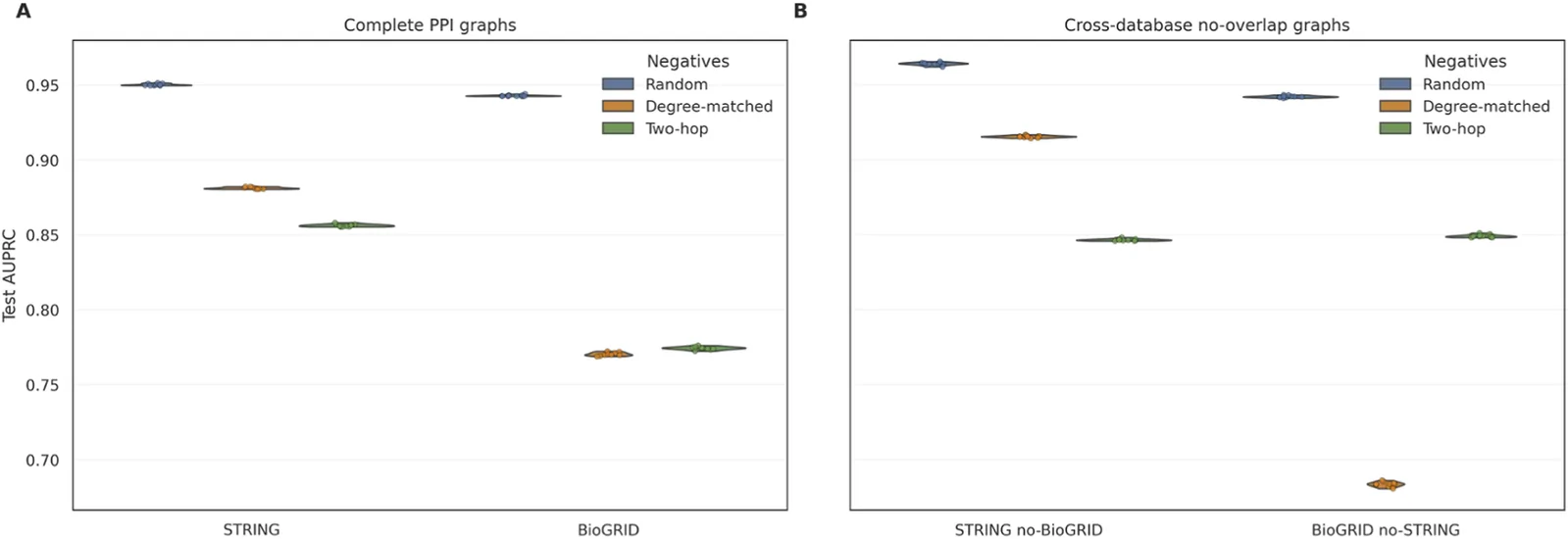
HGB performance distributions across 10 seeds. Violin densities and seed-level points summarize test AUPRC for complete PPI graphs and cross-database no-overlap ablations under random, degree-matched, and two-hop negative-sampling contracts. **(A)** Complete PPI graphs: STRING and BioGRID. **(B)** Cross-database no-overlap graphs: STRING without BioGRID and BioGRID without STRING.

Figure 2B shows the no-overlap ablations and gives a stronger test of whether performance survives cross-resource decontamination. STRING without BioGRID preserves high performance under random negatives (0.964) and degree-matched negatives (0.916), but falls under two-hop negatives (0.847). BioGRID without STRING behaves differently: random negatives produce 0.942, degree-matched negatives drop sharply to 0.684, and two-hop negatives recover to 0.849. This pattern is important because it shows that harder negatives are not interchangeable. Degree matching and two-hop sampling stress different structural assumptions, especially after database overlap is removed.

Figure 3 makes the same result explicit as a paired contrast. In Figure 3A, random-minus-degree-matched differences are positive for all four datasets, confirming that degree matching systematically reduces HGB performance rather than introducing random noise. The largest reduction occurs in BioGRID without STRING (0.259), followed by BioGRID (0.172), STRING (0.069), and STRING without BioGRID (0.049). The ordering indicates that BioGRID-derived tasks are more vulnerable to endpoint-degree control than STRING-derived tasks.

**FIGURE 3:**
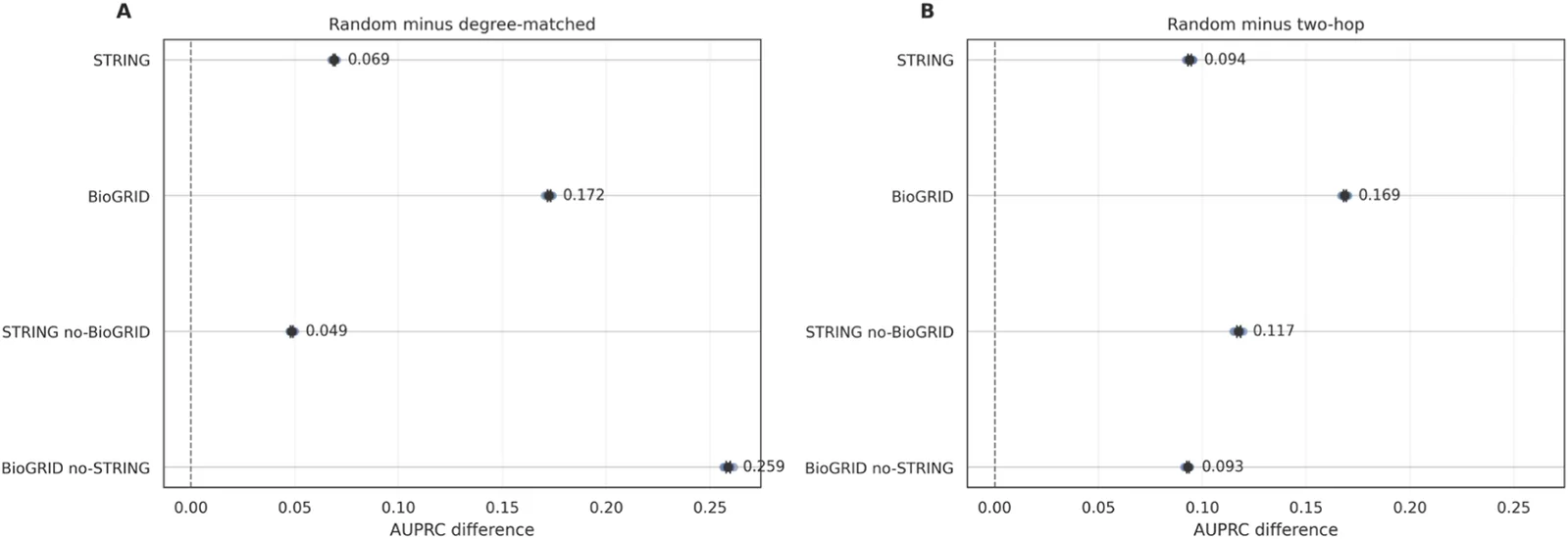
Seed-paired performance change induced by harder negative sampling. Points show per-seed AUPRC differences between random negatives and the harder contracts; whiskers show 95% confidence intervals across 10 seeds. **(A)** Random minus degree-matched AUPRC differences. **(B)** Random minus two-hop AUPRC differences.

Figure 3B compares random negatives with two-hop negatives. The reductions remain positive, but the order changes: BioGRID drops by 0.169, STRING without BioGRID by 0.117, STRING by 0.094, and BioGRID without STRING by 0.093. Compared with Figure 3A, this panel shows that two-hop negatives are especially disruptive for STRING-derived tasks and less disruptive than degree matching for BioGRID without STRING. Together, Figures 2, 3 support the benchmark’s central claim: BioGraphBench-PPI is reproducible across seeds, but sensitive enough to expose when the evaluation protocol is too easy.

### 3.3 Calibration and computational cost distinguish supervised baselines

Figure 4 analyzes model-family behavior one dataset at a time, including the two-layer GCN link-prediction baseline as a direct neural comparator. In Figure 4A, STRING shows a smooth degradation for the structural and embedding baselines from random to harder negatives, with HGB and RF remaining near the top. The GCN follows a different regime: it shows moderate signal under random negatives (AUPRC 0.535) and falls to chance-level performance under degree-matched and two-hop negatives (0.500 and 0.501). In Figure 4B, BioGRID shows a similar neural pattern: HGB and RF remain competitive under harder negatives, whereas the GCN moves from 0.526 under random negatives to approximately 0.500 under both stricter contracts.

**FIGURE 4:**
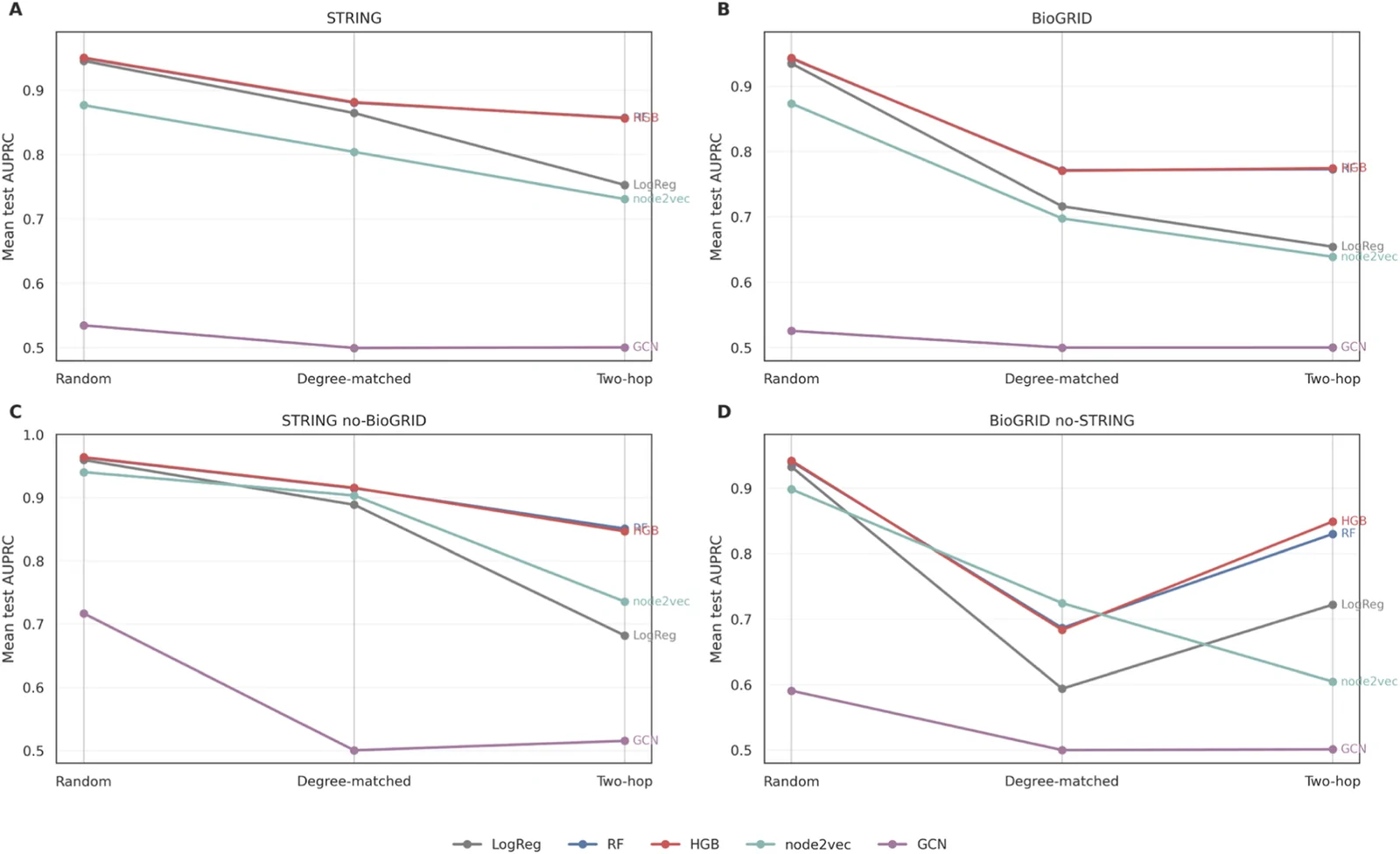
Model-family profiles across negative-sampling regimes. Mean test AUPRC across 10 seeds is shown for logistic regression, random forest, HGB, the node2vec-compatible random-walk baseline, and the two-layer GCN link-prediction baseline in each PPI task **(A)** STRING. **(B)** BioGRID. **(C)** STRING without BioGRID. **(D)** BioGRID without STRING.

Figures 4C,D are the most informative panels because they use no-overlap ablations. In STRING without BioGRID (Figure 4C), HGB and RF are nearly indistinguishable under random and degree-matched negatives, and RF slightly exceeds HGB under two-hop negatives (0.851 *versus* 0.847). The GCN obtains its strongest result in this panel under random negatives (0.717), but its AUPRC drops by 0.217 under degree-matched negatives and by 0.202 under two-hop negatives. In BioGRID without STRING (Figure 4D), the ranking changes more dramatically: node2vec is strongest under degree-matched negatives (0.725), but HGB becomes strongest under two-hop negatives (0.849). The GCN again declines from random negatives (0.591) to degree-matched and two-hop negatives (0.500 and 0.501), confirming that the neural baseline is also contract-sensitive. These panel-level reversals show that model ranking is conditional on model family, data source, and negative-sampling contract.

Figure 5 adds practical diagnostics that cannot be inferred from AUPRC alone. In Figure 5A, logistic regression has the largest calibration error range among the non-neural baselines, especially under harder negatives, whereas HGB remains tightly concentrated at low ECE-10 values. RF and node2vec occupy intermediate positions, with node2vec showing more calibration error than HGB despite being competitive in selected AUPRC settings. The GCN creates a qualitatively different calibration profile: its ECE-10 remains very high across contracts (approximately 0.486–0.500), indicating that the minimal neural baseline is not only weak under hard negatives but also poorly calibrated. This panel therefore supports reporting calibration as a primary companion to ranking metrics, especially when model families differ in how they transform graph structure into probabilities.

**FIGURE 5:**
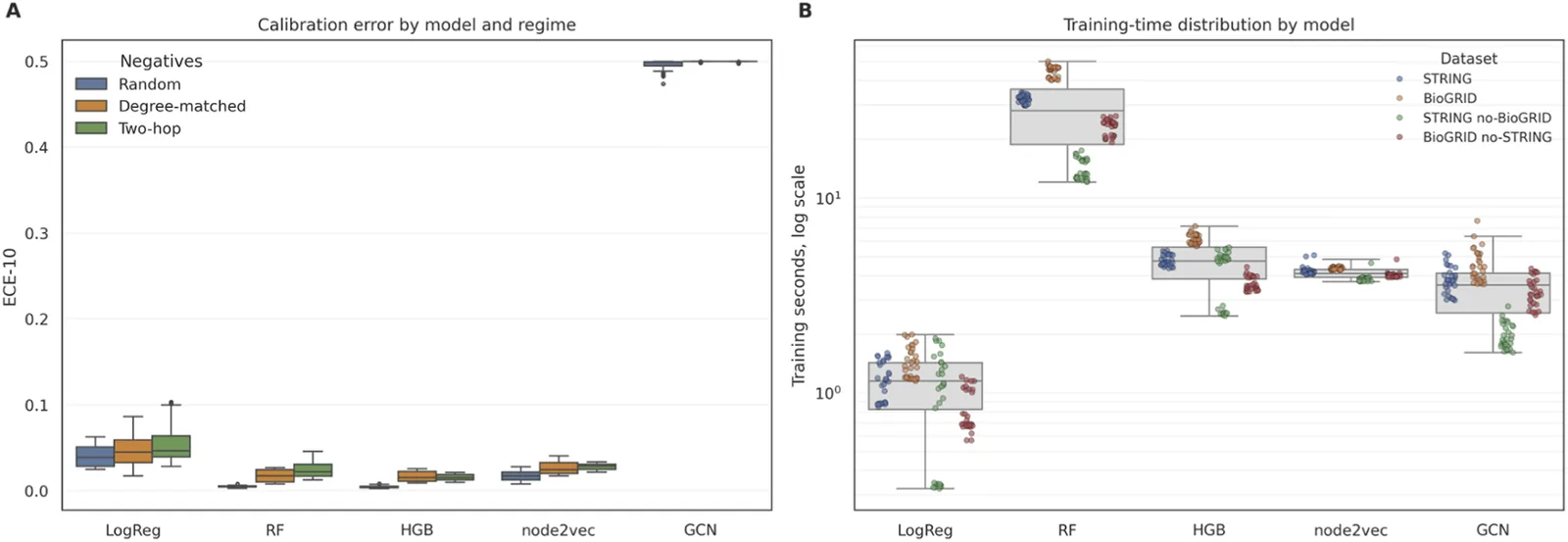
Calibration and computational-cost diagnostics. **(A)** ECE-10 distributions by model family and negative-sampling contract. **(B)** Training-time distributions by model family, with points showing dataset-level runs.

Figure 5B shows that runtime separates the baselines in a way that matters for benchmark adoption. Logistic regression is the fastest and most variable mainly because its cost scales with feature-table size. RF has the highest training-time distribution, reflecting the cost of ensemble fitting across large split files. HGB is less expensive than RF while retaining strong AUPRC and calibration, and node2vec has a compact runtime band because the random-walk embedding stage dominates its cost profile. The GCN occupies a moderate CPU-runtime range in this implementation, with mean training times of approximately 1.8–5.3 s depending on graph and contract, partly because training was capped at 100,000 positive and 100,000 sampled-negative pairs per seed. The practical conclusion is that HGB remains a strong default baseline, but RF, node2vec, and GCN are all necessary comparators because they represent distinct accuracy, calibration, and cost trade-offs.

### 3.4 Functional node classification remains difficult

The multilabel OBNB BioGRID + GOBP task remains in the manuscript as a secondary stress test rather than as part of the main PPI claim (Table 5). Its performance regime is deliberately different. A constant-feature logistic regression control gave the chance-level macro-AUROC of 0.500 and macro-AUPRC 0.0107. One-hot log-degree features raised logistic-regression macro-AUROC to 0.531 and macro-AUPRC to 0.0144, while the pilot MLP and GCN did not produce a decisive improvement. Because this experiment evaluates node classification rather than PPI link prediction, it is interpreted only as evidence that BioGraphBench can expose harder biological tasks with different metric behavior.

**TABLE 5:** Test performance on OBNB BioGRID + GOBP multilabel node classification. Micro metrics use per-label thresholds selected on validation data. The constant control did not have a separate threshold-tuning record, so precision and recall are not reported.

Model	Feature	Macro-AUROC	Macro-AUPRC	Micro-F1	Precision	Recall
Constant logistic regression	Constant	0.500000	0.010685	0.021478	—	—
Logistic regression	One-hot log-degree	0.530988	0.014365	0.025313	0.013053	0.416216
MLP	One-hot log-degree	0.488016	0.012944	0.021706	0.011027	0.687838
GCN	One-hot log-degree	0.493559	0.015926	0.021249	0.010814	0.605405

Figure 6 should be read as a secondary stress-test figure rather than as part of the main PPI performance claim. In Figure 6A, macro-AUROC and macro-AUPRC remain low across the constant control, logistic regression, MLP, and GCN, showing that sparse GOBP annotation cannot be recovered effectively from simple degree-bin information or the pilot neural models. The contrast with Figures 2–4 is intentional: strong structural separability in PPI link prediction does not transfer automatically to multilabel functional annotation.

**FIGURE 6:**
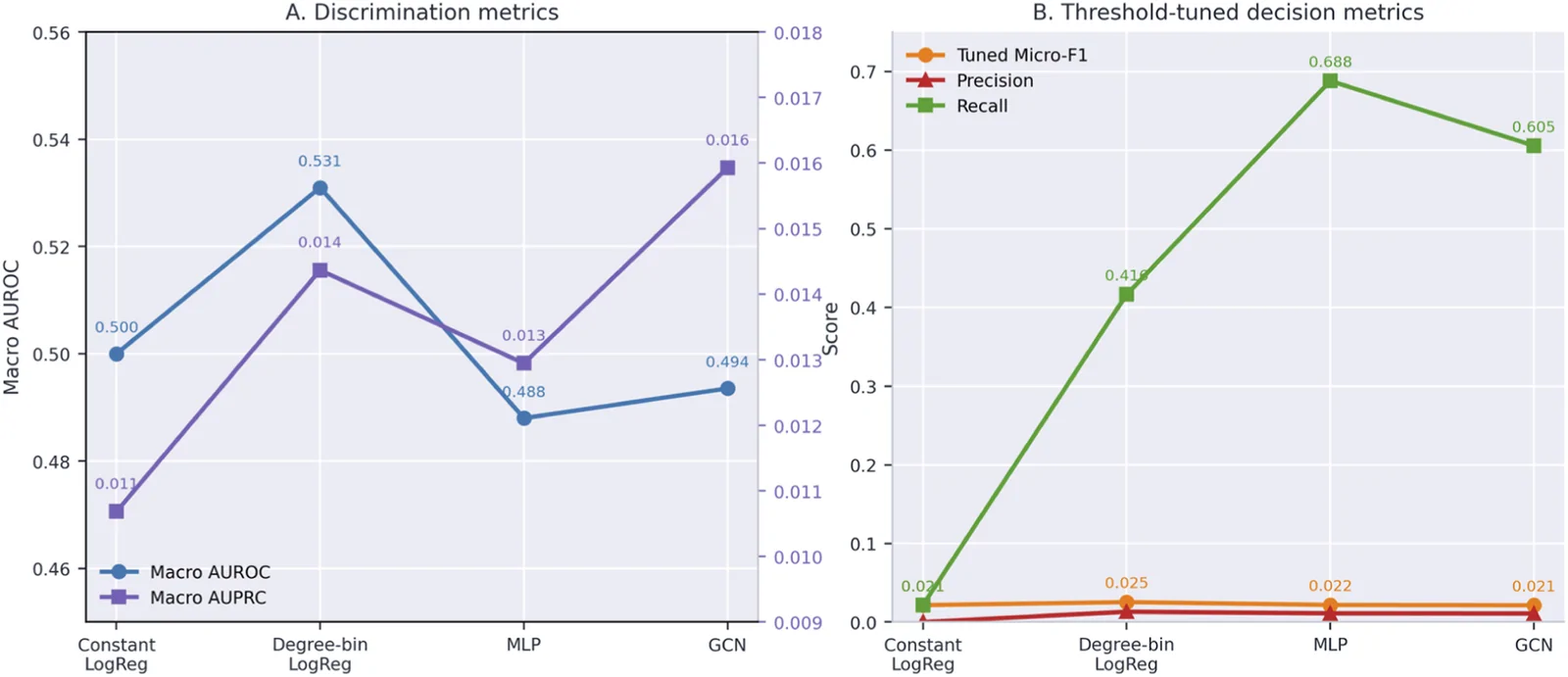
OBNB BioGRID + GOBP node-classification trade-offs. **(A)** Test macro-AUROC and macro-AUPRC. **(B)** Test micro-F1 and, where available, precision and recall; validation-selected per-label thresholds were used for the one-hot degree logistic regression, MLP, and GCN models.

Figure 6B shows the decision-level failure mode. Threshold tuning produces non-zero micro-F1, but the gains come with very low precision. Logistic regression obtains the highest micro-F1 (0.0253) by balancing low precision (0.0131) against moderate recall (0.4162), while MLP and GCN increase recall but do not solve the precision problem. This figure therefore supports a bounded interpretation of OBNB: it is useful evidence that BioGraphBench can expose harder biological tasks, but it is interpreted as a secondary stress test rather than as part of the central PPI link-prediction claim.

### 3.5 Result-derived requirements for benchmark interpretation

The integrated results define empirical requirements for subsequent model submissions because the benchmark exposes three separable sources of evidence: task scale, contract sensitivity, and model-family behavior. Table 1 and 2 show that the central contribution is not a small illustrative example but a reproducible PPI test bed composed of four auditable link-prediction tasks. The two complete graphs contain 18,767–20,376 nodes and 738,805–961,531 canonical edges, while the no-overlap ablations retain 314,539–538,003 edges. This scale is large enough for structural shortcuts to emerge and large enough for runtime and calibration to matter, but still compact enough for repeated multi-seed evaluation on local CPU hardware.

The first empirical requirement is that any submitted model must be interpreted across sampled-nonedge contracts rather than under a single negative rule. Table 3 shows that random negatives produce very high HGB AUPRC values, ranging from 0.942 to 0.964, with narrow confidence intervals across 10 seeds. Those values are reproducible, but they are also the easiest condition. When the same datasets are evaluated with degree-matched negatives, HGB AUPRC falls to 0.684–0.916, and the largest random-to-degree reduction reaches 0.259 in the BioGRID-without-STRING ablation. This is not random instability; it is a systematic change in task definition. Therefore, a future result reported only under random negatives would be incomplete even if it were numerically high.

The second requirement is direct comparison against strong non-GNN floors. The benchmark shows that supervised structural baselines are not weak controls. HGB and RF remain competitive across the most important PPI conditions, and their seed-to-seed variation is small. Figure 2 supports this point by showing tight HGB distributions across the 10 seeds, while Figure 3 shows that the same stable model undergoes large paired drops when the negative contract changes. In other words, the benchmark is stable enough to audit model behavior but sensitive enough to reveal when performance depends on an overly permissive sampling rule.

The third requirement is that calibration and computational cost must accompany ranking metrics. Figure 5A shows that strong AUPRC does not imply equally strong probability quality: logistic regression can approach tree-based ranking under easier regimes while producing larger ECE-10 values, whereas HGB combines high AUPRC with low calibration error in the random-negative condition. The GCN extends this point because its probability outputs remain poorly calibrated even when its random-negative AUPRC is above chance in selected no-overlap tasks. Figure 5B adds a practical constraint. RF is computationally heavier, HGB occupies a more favorable accuracy-cost region, node2vec introduces a distinct random-walk embedding cost, and the capped CPU GCN baseline occupies an intermediate runtime band while providing limited hard-negative discrimination. These results justify reporting Brier score, ECE-10, training time, and inference time alongside AUROC and AUPRC.

The fourth requirement concerns neural link-prediction models. The GCN baseline was evaluated under the same four PPI graphs, three sampled-nonedge contracts, 10 seeds, and metric set used for the retained structural and embedding baselines. Its behavior strengthens the benchmark claim: message passing over the training-positive graph did not remove contract sensitivity. Instead, the GCN performed best under random negatives in STRING without BioGRID (AUPRC 0.717) and BioGRID without STRING (AUPRC 0.591), while degree-matched and two-hop negatives drove performance close to chance. A neural model submitted to BioGraphBench-PPI must therefore be interpreted against the same non-GNN floors and the same negative-sampling regimes, not only against a single random-negative split.

## 4 Discussion

BioGraphBench-PPI shows that the scientific value of a biomedical graph benchmark depends on the interpretability of its experimental contract. The integrated tables and figures support a single coherent conclusion: high PPI link-prediction performance under random negatives is real and reproducible, but incomplete. Degree-matched and two-hop contracts change both the absolute level of performance and the relative ranking of model families. This means the benchmark is not simply measuring model capacity; it is also measuring the assumptions embedded in task construction.

The overlap analysis makes the same point at the data level. STRING and BioGRID shared 423,528 mapped protein pairs, so a cross-resource evaluation without alignment could mistake a previously observed interaction for independent evidence. Removing direct overlaps did not eliminate high performance under random sampled nonedges, indicating that substantial structural signal remained after direct pair removal. The ablations therefore reduce one explicit leakage path while revealing that higher-order topology remains highly predictive. They cannot remove common experimental literature, node composition, or indirect topology, and should be interpreted as partial independence controls rather than as proof of fully independent biological evidence.

Calibration is a second central contribution. Biomedical prediction scores may be used to prioritize expensive laboratory work, so the probability attached to a candidate can matter independently of its rank. In the present experiments, logistic regression sometimes approached the ranking performance of tree ensembles while producing substantially larger ECE. Under random sampled nonedges, HGB achieved AUPRC values of 0.942–0.964 with ECE-10 below 0.0061. This result supports a benchmark requirement that discrimination and calibration be reported together. It also motivates calibration curves and decision-focused evaluation in future releases, especially under more realistic class prevalence.

The retained functional node-classification task prevents the benchmark from being interpreted as a single easy PPI exercise. Its sparse GOBP labels were not recovered effectively from degree bins or by the pilot GCN, confirming that different biological graph tasks expose different failure modes. The PPI HGB and OBNB GCN results answer different questions, use different prediction units, labels, and metrics, and therefore are not direct model-family comparisons. In the present manuscript, OBNB is a secondary stress test rather than a fully developed benchmark family.

The present findings are consistent with prior work showing that baseline strength, data splits, and evaluation design can materially affect model comparisons in graph learning (Shchur et al., 2018; Dwivedi et al., 2023). BioGraphBench-PPI extends this observation by quantifying how uniform random, degree-matched, and two-hop sampled nonedges alter evaluation difficulty and model ranking within open PPI resources.

The design also complements recently published network-bioinformatics tools. Protein Contact Network Explorer demonstrates the value of interpretable topology and explicit validation, NOVAGene emphasizes accessible network optimization and visualization, and Exp2Ipynb treats reproducibility as a workflow property rather than an afterthought (Liebal et al., 2021; Bacong et al., 2026; Ganapathy et al., 2026). BioGraphBench extends these priorities to predictive benchmarking by binding each score to source provenance, a split contract, a feature policy, and machine-readable outputs. This positioning directly supports methods, tools, benchmarking suites, and provenance-aware workflows in network bioinformatics.

Several limitations constrain the current conclusions. First, the present study is intentionally scoped to PPI link prediction; broader network-bioinformatics tasks require additional task-specific validation. Second, the two-hop strategy is a local hard-negative proxy rather than a complete community-aware or temporally held-out evaluation. Third, the node2vec-compatible baseline is auditable but not exhaustively tuned. Fourth, the GCN experiment is intentionally simple: it uses structural degree-derived node features and a dot-product decoder, so it should be interpreted as a minimal neural baseline rather than as an exhaustive GNN search. More expressive GraphSAGE, GAT/GIN, and SEAL-style subgraph methods remain important extensions under the same audit contract.

The next benchmark release should extend the present PPI protocol with competitive GNN link-prediction models, robustness tests under edge perturbation and STRING-threshold variation, model interpretability, and scalability measurements for memory, parameters, and inference time. Those extensions should preserve the same audit-first contract rather than replacing it with a model-centered leaderboard.

## 5 Conclusion

BioGraphBench-PPI converts open protein-interaction resources into reconstructible experimental tasks and exposes a central methodological result: PPI link-prediction conclusions depend strongly on the sampled-nonedge contract. Random negatives produced high and stable structural baselines, whereas degree-matched and two-hop negatives altered task difficulty and, in several settings, model ranking. These effects persisted when a two-layer GCN was evaluated under the same four PPI tasks, three sampled-nonedge contracts, and multi-seed protocol, showing that the benchmark can expose contract-dependent behavior across both structural and neural baselines.

The resulting benchmark is not a claim that PPI prediction is solved. Rather, it provides an auditable framework in which model performance can be interpreted against explicit data provenance, leakage controls, negative-sampling rules, strong non-GNN baselines, calibration, statistical uncertainty, and computational cost. Future extensions should evaluate more expressive GNN architectures and additional robustness conditions under the same experimental contract, while preserving the public and reproducible evidence chain established here.

## Data Availability

The original contributions presented in the study are included in the article/supplementary material, further inquiries can be directed to the corresponding author.
